# Cost effectiveness of immune checkpoint inhibitors for treatment of non-small cell lung cancer: A systematic review

**DOI:** 10.1371/journal.pone.0238536

**Published:** 2020-09-02

**Authors:** Haiying Ding, Wenxiu Xin, Yinghui Tong, Jiao Sun, Gaoqi Xu, Ziqi Ye, Yuefeng Rao

**Affiliations:** 1 Department of Pharmacy, Cancer Hospital of the University of Chinese Academy of Sciences (Zhejiang Cancer Hospital), Institute of Cancer and Basic Medicine (IBMC), Chinese Academy of Sciences, Hangzhou, China; 2 Department of Pharmacy, The First Affiliated Hospital, College of Medicine, Zhejiang University, Hangzhou, China; URCEco Ile de France Hopital de l’Hotel Dieu, FRANCE

## Abstract

**Background:**

Immune checkpoint inhibitors (ICIs) for treatment of non-small cell lung cancer (NSCLC) have been rapidly evolving. ICIs are likely to be more effective but also lead to escalating healthcare costs.

**Objectives:**

The aim of this study was to evaluate the cost effectiveness of immune checkpoint inhibitors (ICIs) for treatment of non-small cell lung cancer (NSCLC).

**Methods:**

We searched the PubMed, Web of Science, and Cochrane Library for studies comparing the cost effectiveness of ICIs for NSCLC. Potential studies identified were independently checked for eligibility by two authors, with disagreement resolved by a third reviewer. Quality of the included studies was evaluated using Consolidated Health Economic Evaluation Reporting Standards checklists.

**Results:**

A total of 22 economic studies were included. Overall reporting of the identified studies largely met CHEERS recommendations. In the first-line setting, for advanced or metastatic NSCLC patients with PD-L1 ≥ 50%, pembrolizumab appeared cost-effective compared with platinum-based chemotherapy in the US and Hong Kong (China), but not in the UK and China. The cost-effectiveness of pembrolizumab versus chemotherapy for first-line treatment of NSCLC in PD-L1 ≥ 1% patients remained obscure. Regardless of PD-L1 expression status, pembrolizumab in combination with chemotherapy could be a cost-effective first-line therapy in the US. On the contrary, addition of atezolizumab to the combination of bevacizumab and chemotherapy was not cost-effective for patients with metastatic non-squamous NSCLC from the US payer perspective. In the second-line setting compared with docetaxel, pembrolizumab was cost-effective; though nivolumab was not cost-effective in the base case, it could be by increased PD-L1 threshold. Results of the cost-effectiveness of atezolizumab second-line treatment remained inconsistent. In addition, the adoption of durvalumab consolidation therapy after chemoradiotherapy could be cost-effective versus no consolidation therapy for patients with stage III NSCLC.

**Conclusions:**

Immunotherapy can be a cost-effective option for treatment of NSCLC in several scenarios. A discount of the agents or the use of PD-L1 expression as a biomarker improves the cost-effectiveness of immunotherapy.

## Introduction

Lung cancer is the most common cancer and the leading cause of cancer mortality worldwide, with an estimated incidence of more than 2 million cases and approximately 1.8 million deaths [1]. Non-small cell lung cancer (NSCLC) accounts for 80–90% of lung cancer. Up to approximately 55% of cases are diagnosed at a metastatic stage, which leads to poor long-term prognosis [2].

Patients with targetable genetic aberrations gain significant benefits from targeted therapies; however, the subsets represent a small fraction of patients with NSCLC [3]. Over a long period in the past, the majority of patients without an identified molecular subtype relied primarily on traditional chemotherapy with modest improvement in outcome. The recent introduction of immune checkpoint inhibitors (ICIs), namely monoclonal antibodies directed against programmed death receptor 1 (PD-1), programmed death ligand 1 (PD-L1) and cytotoxic T-cell lymphocyte antigen-4 (CTLA-4) monoclonal antibodies, has resulted in an increase in overall survival (OS) rates of patients with advanced NSCLC on the basis of numerous clinical trials [4–10]. Therefore, ICIs have become the standard of care in appropriate clinical circumstances for patients with NSCLC.

Given the rising economic burden of cancer care, for example, cancer care in the United States is expected to reach $173 billion by 2020, cost is becoming an increasingly critical consideration in cancer care other than clinical benefit and toxicity [11]. Cost-effectiveness analysis is an important strategy to assess whether new interventions provide clinical benefit at a reasonable cost, which has major implications on health policy and public policy [12, 13]. As such, evaluating the cost effectiveness of new and expensive oncology therapies is of great concern.

ICIs represent the fastest growing part of the oncology therapeutics market, heightening the need for economic evaluation of these novel agents [14, 15]. Verma V. et al. conducted a systematic review to evaluate the economic impact of ICIs, and analyzed studies of head/neck, lung, genitourinary, and melanoma malignancies treated with ICIs, demonstrating that pembrolizumab was cost-effective for NSCLC while nivolumab was not [16]. However, plenty of new cost-effectiveness investigations associated with ICIs for treatment of NSCLC have been published along with constantly updated ICIs clinical trials, which could substantially alter the above-mentioned conclusions.

### Objectives

As expansion in the availability of immune checkpoint inhibitors in NSCLC continues, the aim of this review was to make an overview of the currently available literature on cost-effectiveness of immune checkpoint inhibitors in treatment of NSCLC and to satisfy the need of decision makers to maximize benefits under resource constraints.

## Methods

### Databases and search strategy

The systematic review was performed following the Preferred Reporting Items for Systematic Reviews and Meta-Analyses (PRISMA) guidelines (S1 File) [17]. We searched the PubMed, Web of Science, Cochrane CENTRAL databases in December of 2019. The main search terms included non-small cell lung cancer, immunotherapy, immune checkpoint inhibitor, PD-1, PD-L1, cost, and economic. Based on guidelines for management of NSCLC, we also included the following agents in the search: pembrolizumab, nivolumab, atezolizumab, ipilimumab and durvalumab. No language or date restrictions were initially imposed. The detailed search strategy is shown in the S2 File. Moreover, references cited in the identified studies, recent review articles and other relevant studies were also scrutinized to identify potentially relevant articles that may have been missed in the original search. Unpublished abstracts were not included due to the inability to fully evaluate validity and methodologies. We de-duplicated the identified results using EndNote^®^ (Clarivate Analytics) [18].

### Eligibility criteria

Studies were eligible for review if they performed a cost-effectiveness analysis (CEA) or cost-utility analysis (CUA) that assessed the immune checkpoint inhibitors for NSCLC. The related immune checkpoint inhibitors for NSCLC include anti-PD-1 agents (nivolumab and pembrolizumab), anti-PD-L1 agents (atezolizumab and durvalumab) and anti-CTLA-4 agents (ipilimumab). Studies were excluded if they (1) assessed only the cost or effectiveness of therapeutic regimens; (2) did not report the effectiveness as quality-adjusted life year (QALY); (3) absence of a report summarizing statistical result, such as an incremental cost-effectiveness ratio (ICER); (4) were not written in English; or (5) were case reports, letters, news, comments, editorials, conference abstracts, or systematic reviews.

### Review for inclusion

The titles and abstracts of all potential studies identified were checked for eligibility independently by two investigators. The eligible studies were proceeded to a full-text review by the two investigators to finalize study selection. Disagreement between the two investigators were resolved by a third investigator.

### Data extraction and synthesis

Data from the selected studies were extracted and synthesized in Microsoft Excel. Data collected included first author, country, publication year, sponsorship source, study population, study design (type of economic evaluation), study perspective (societal, health provider, payer, etc.), model structure, time horizon, interventions, year of costing, type of currency, source of cost, source of effectiveness, outcome measure, sensitivity analysis, willingness-to-pay threshold, results (total costs, effectiveness and ICER, etc.) and conclusion.

### Quality assessment

Quality assessment was independently performed by two investigators using the CHEERS (Consolidated Health Economic Evaluation Reporting Standards) checklist, which was developed by a task force supported by the International Society for Pharmacoeconomics and Outcomes Research (ISPOR) [19]. The CHEERS checklist, aiming to optimize reporting of health economic evaluations, comprises 24 items which are subdivided into six categories: (1) title and abstract, (2) introduction, (3) methods, (4) results, (5) discussion, and (6) other.

### Data analysis

Total costs, effectiveness (QALYs), and ICERs were compared among the included studies, stratified by treatment line. To make a comparison between the different currencies used in different countries, the reported costs were converted into US dollars (2019) using a web-based tool. In addition, the authors’ conclusions regarding their modeled interventions were reported.

## Results

**Fig 1:**
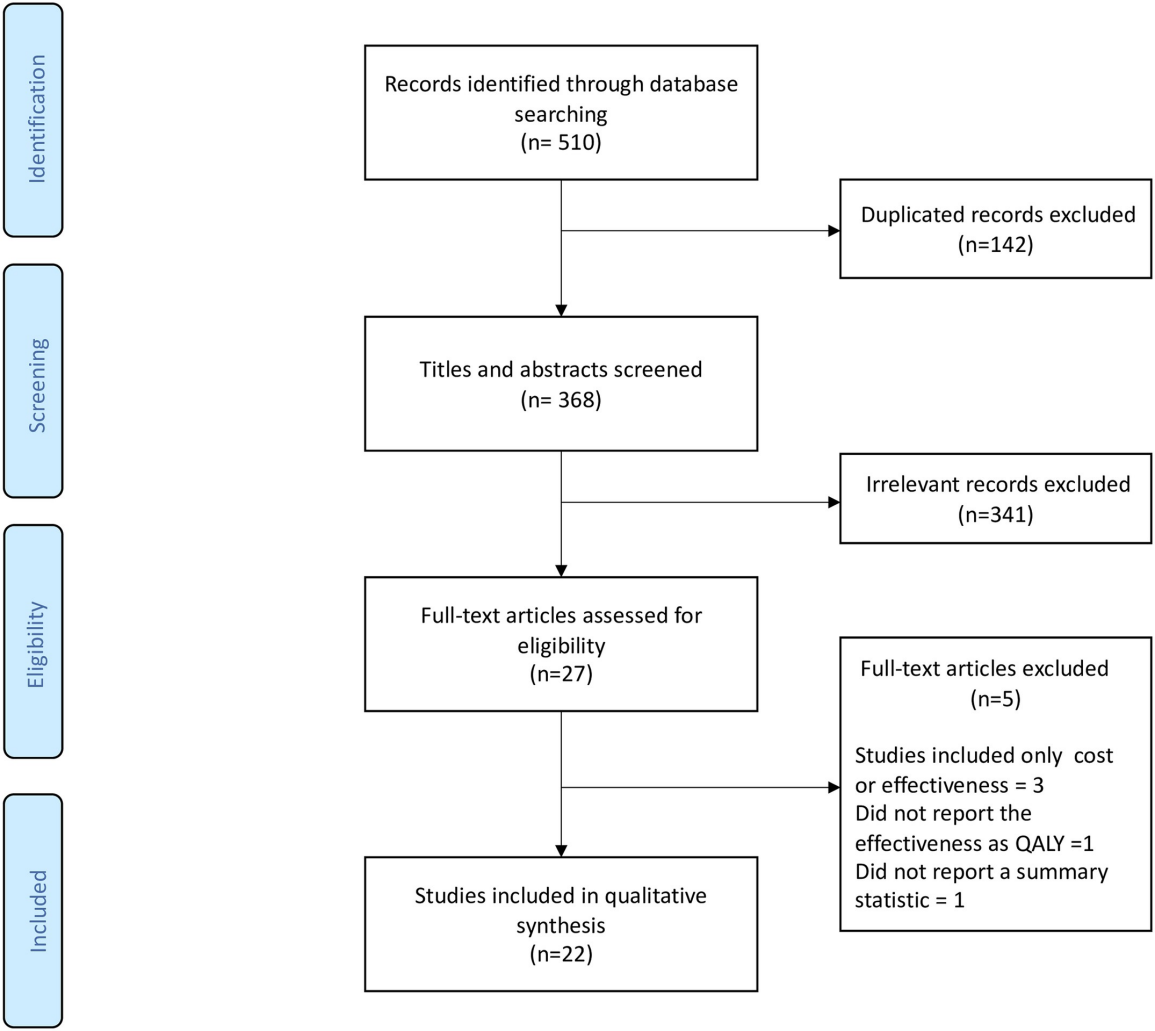
PRISMA flowchart. PRISMA, Preferred Reporting Items for Systematic Reviews and Meta-Analyses.

In the initial search, 510 records were identified from the databases (Fig 1). Following removal of duplicates, titles and abstracts of 368 studies were screened. Of these, 341 irrelevant records were excluded. A total of 27 articles were then fully reviewed and assessed for eligibility. Finally, 22 studies were included in this systematic review [20–41].

### Characteristics of the included studies

The characteristics of all included studies are presented in S1 Table. Most of the studies were conducted in 2019 [28–41]. Studies were conducted from countries all over the world, with ten from the USA [22–27, 29, 30, 32, 33], seven from China (one from HongKong) [34–36, 38–41], two from Canada [20, 37], and one each from Australia [31], France [28] and Switzerland [21]. Most studies were assessed from the perspective of health care system (N = 12) [20–22, 25, 26, 28–31, 35, 37, 40], some used a payer perspective (N = 8) [23, 24, 27, 32, 33, 38, 39, 41], and the remainder used a perspective of society (N = 1) [34] and hospital authority (N = 1) [36].

The majority of the studies (N = 14) [23, 25–28, 30, 32–34, 36, 38–41] analyzed the cost-effectiveness of ICIs in the first-line treatment of NSCLC, of which one study was for patients with squamous cell carcinoma (SCC) and four studies were for non-SCC, while 7 studies [20–22, 24, 31, 35, 37] in second-line treatment (one study for SCC and two studies for non-SCC) and one study in stage III NSCLC after chemoradiotherapy [29]. Treatments compared in each study varied across studies. Among studies analyzing the first-line treatment, most studies compared pembrolizumab with platinum-based chemotherapy based on KEYNOTE 024 [9] or KEYNOTE 042 trial [42] (N = 10) [23, 25, 26, 28, 32, 34, 36, 38, 40, 41]. The remainders evaluated pembrolizumab plus chemotherapy versus chemotherapy alone or pembrolizumab alone, combination of atezolizumab and chemotherapy versus chemotherapy. As for second-line treatment, the majority of the studies assessed nivolumab versus docetaxel (N = 4). In total, the most commonly modeled intervention was pembrolizumab (N = 13), followed by nivolumab (N = 5), atezolizumab (N = 3), durvalumab (N = 1) and a mix of treatment options (N = 1).

All of the included studies built economic models to assess the cost-effectiveness of ICIs. Of these, ten studies used Markov model, five studies used a partitioned survival model and two studies adopted both. Other models included decision-analytic model, cohort simulation model and microsimulation model. The time horizon evaluated by the studies varied significantly. Most studies evaluated a time horizon of lifetime, and the shortest time horizon included was 5 years in the study assessing durvalumab consolidation therapy, whereas one study did not report the time horizon of analysis.

### Cost-effectiveness outcomes

Table 1 summarizes the outcomes of the included studies according to treatment line (first-line, second-line and locally advanced cancer) and PD-L1 expression level (Table 1).

**Table 1:** Overview of the outcomes of the included studies.

Study, year	Costs	QALYs	ICER (per QALY gained)	WTP threshold (per QALY gained)	Conclusions
**First-line (PD-L1 ≥ 50%) (n = 6)**					
Huang et al., 2017 [23]	Pembro: $362663 PBC: $260,233	Pembro: 2.60 PBC: 1.55	Pembro vs PBC: $97,621	$100,000; $150,000	Pembro is cost-effective compared with PBC from the US third-party public health care payer perspective.
Georgieva et al., 2018 [25]	Perspective of British NHS: No dependency: pembro: $99,000 PBC: $34,000 Moderate dependency: pembro: $121,000 PBC: $38,000 Perspective of US cost: No dependency: pembro: $132,000 PBC: $73,000; Moderate dependency: pembro: $160,000 PBC: $81,000	No dependency: pembro: 3.06 PBC: 1.11 Moderate dependency: pembro: 2.69 PBC: 1.06	pembro vs PBC UK perspective: No dependency: $34,000; Moderate dependency: $52,000 US perspective: No dependency: $31,000; Moderate dependency: $49,000	UK: £30,000 ($42,048); US: $100,000	Pembro is cost-effective compared with PBC in the US but not the UK.
Hu et al., 2018 [26]	Pembro: £92,833 ($121,760) PBC: £20,368 ($26,711)	Pembro: 1.554 PBC: 0.71	Pembro vs PBC: £86,913 ($113,978)	£50,000 ($65,570)	Pembro is not cost-effective at its current list price and a discount of 50% or more is required for it to be cost-effective comparing to PBC from the UK health care perspective.
Chouaid et al., 2019 [28]	SCC: pembro: €125,261 ($140,405) PBC: €63,229 ($70,873) Non-SCC: PBC: €70,790 ($79,348) PBC with gem plus bev: €74,042 ($82,994) PBC with ptx plus bev: €80,330 ($90,042) PBC with pem: €86,902 ($97,408) pembro: €133,966 ($150,162) PBC with pem plus bev: €148,913 ($166,917)	SCC: pembro: 1.57 PBC: 0.83 Non-SCC: PBC: 1.04 PBC with gem plus bev: 1.04 PBC with ptx plus bev: 1.38 PBC with pem: 1.23 pembro: 2.06 PBC with pem plus bev: 1.42	Relative to PBC SCC: pembro: €84,097 ($94,264) Non-SCC: PBC with gem plus bev: strictly dominated PBC with ptx plus bev: €28,448 ($31,887) PBC with pem: strictly dominated Pembro: €78,729 ($88,247) PBC with pem plus bev: strictly dominated	€100,000 ($112,090)	Pembro appears cost-effective versus chemotherapy from the perspective of French healthcare system.
Liao et al., 2019 [34]	Pembro: $115,019 PBC: $68,657	Pembro: 1.10 PBC: 0.65	Pembro vs PBC: $103,128	$26,481	Pembro is not likely to be cost-effective from the perspective of Chinese society.
Loong et al., 2019 [36]	Pembro: HK$1147,792 ($147,342) PBC: HK$898,715 ($115,368)	Pembro: 1.69 PBC: 1.41	Pembro vs PBC: HK$ 865,189 ($111,064)	HK$1017,819 ($130,657)	Pembro in a BTS to identify patients with PD-L1≥ 50% can be considered cost effective compared with PBC from the perspective of Hospital Authority in Hong Kong (China).
**First-line (PD-L1 ≥ 1%) (n = 4)**					
Huang et al., 2019 [32]	PD-L1≥1%: pembro: $230,954 PBC: $167,046 PD-L1 ≥ 50%: NR PD-L1 ≥1% and <49%: NR	PD-L1≥1%: pembro: 1.77 PBC: 1.28 PD-L1 ≥ 50%: pembro vs PBC: 0.77 PD-L1 ≥1% and <49%: pembro vs PBC: 0.28	Pembro vs PBC: PD-L1 ≥1%: $130,155 PD-L1 ≥ 50%: $111,781 PD-L1 ≥1% and <49%: $161,546	$150,000	Pembro is cost-effective versus PBC in patients with PD-L1 ≥ 1%, but not in the subgroup with PD-L1 1~49% from the US third-party public healthcare payer perspective.
She et al., 2019 [38]	PD-L1 ≥ 50%: pembro: $261,848 PBC: $175,684 PD-L1 ≥ 20%: pembro: $249,065 PBC: $174,503 PD-L1≥1%: pembro: $239,205 PBC: $168,318	PD-L1 ≥ 50%: pembro: 2.10 PBC: 1.47 PD-L1 ≥ 20%: pembro: 1.93 PBC: 1.47 PD-L1 ≥ 1%: pembro: 1.83 PBC: 1.44	Pembro vs PBC: PD-L1 ≥ 50%: $136,229 PD-L1 ≥ 20%: $160,626 PD-L1 ≥ 1%: $179,530	$150,000	Pembro is cost-effective compared with PBC in patients with PD-L1 ≥ 50%, but not in the PD-L1 ≥ 20% and 1% populations from the US payer perspective.
Weng et al., 2019 [40]	PD-L1 ≥ 50%: pembro: $117,390 PBC: $63,605; PD-L1 ≥ 20%: pemro: $112,341 PBC: $64,862 PD-L1 ≥ 1%: pemro: $104,747 PBC: $64,919	PD-L1 ≥ 50%: pembro: 1.87 PBC: 0.74 PD-L1 ≥ 20%: pembro: 1.78 PBC: 0.77 PD-L1 ≥ 1%: pembro: 1.37 PBC: 0.78	Pembro vs PBC: PD-L1 ≥ 50%: $47,596 PD-L1 ≥ 20%: $47,184 PD-L1 ≥ 1%: $68,061	$180,000	Pembro is cost-effective compared with PBC with all PD-L1 expression level (≥50%, ≥20%, and ≥1%) from the US health care perspective.
Zhou et al., 2019 [41]	PD-L1 ≥ 50%: pembro: $95,168 PBC: $29,846 PD-L1 ≥ 20%: pembro: $81,867 PBC: $30,671 PD-L1 ≥ 1%: pembro: $73,615 PBC: $29,482	PD-L1 ≥ 50%: pembro: 2.81 PBC: 1.02 PD-L1 ≥ 20%: pembro: 2.28 PBC: 1.07 PD-L1 ≥ 1%: pembro: 2.16 PBC: 1.04	Pembro vs PBC: PD-L1 ≥ 50%: $36,493 PD-L1 ≥ 20%: $42,311 PD-L1 ≥ 1%: $39,404	$26,508	Pembro is not cost-effective compared with PBC regardless of PD-L1 expression (≥50%, ≥20%, and ≥1%) from the perspective of payers in China.
**First-line (all PD-L1 expression levels) (n = 4)**					
Insinga et al., 2018 [27]	PD-L1≥50%: pembro+chemo: $376,584 pembro: $203,358 chemo: $183,810 PD-L1 1–49%: pembro+chemo: $343,216 chemo: $209,545 PD-L1 < 1%: pembro+chemo: $251,192 chemo: $168,140	PD-L1≥50%: pembro+chemo: 3.24 pembro: 2.06 chemo: 1.37 PD-L1 1–49%: pembro+chemo: 3.47 chemo: 1.47 PD-L1 < 1%: pembro+chemo: 1.89 chemo: 1.44	Pembro+chemo vs chemo PD-L1≥50%: $103,402 PD-L1 1–49%: $66,837 PD-L1 < 1%: $183,529 Pembro+chemo vs pembro PD-L1≥50%: $147,365	$180,000	Pembro+chemo is cost-effective in previously untreated non-squamous NSCLC patients with PD-L1≥1% from the US third-party healthcare payer perspective.
Criss et al., 2019 [30]	Base Case 1: BCP: $112,551 ABCP: $244,166 Base Case 2: CP: $82,738 BCP: $112,551 ABCP: $244,166 pembro+CP: $226,282	Base Case 1: BCP:1.48 ABCP: 2.13 Base Case 2: CP: 1.11 BCP: 1.48 ABCP: 2.13 pembro+CP: 2.45	Base Case 1: ABCP vs BCP: $201,676 Base Case 2: BCP vs CP: $80,671 ABCP vs pembro+CP: dominated pembro+CP vs BCP: $116,698	$100,000	Atezo combination is not cost-effective compared with BCP and provided suboptimal incremental benefit compared with cost vs pembro combination from the perspective of US health care sector.
Insinga et al., 2019 [33]	Pembro+chemo: $231,209 Chemo: $111,758	Pembro+chemo: 2.80 Chemo: 1.41	Pembro vs chemo: $86,293	$100,000	Pembro+chemo can be a cost-effective first-line treatment for metastatic squamous NSCLC patients for whom chemotherapy is currently administered from the US third-party healthcare payer perspective.
Wan et al., 2019 [39]	ABCP: $389,550 BCP: $154,552 TC: $8,434	ABCP: 1.390 BCP: 0.977 TC:0.652	ABCP Vs BCP: $568,967 ABCP Vs TC: $516,114 BCP Vs TC: $449,029	$100,000	ABCP is not cost-effective compared with BCP or TC for patients with metastatic non-squamous NSCLC from the US payer perspective.
**Second-line (n = 7)**					
Goeree et al., 2016 [20]	Markov model: nivo: $139,016 doc: $38,812 erl: $39,920 PS model: nivo: $141,973 doc: $38,029 erl: $40.329	Markov model: nivo: 1.23 doc: 0.58 erl: 0.54 PS model: nivo: 1.24 doc: 0.59 erl: 0.55	Markov model: nivo vs doc: $152,229 nivo vs erl: $141,838 PS model: nivo vs doc: $151,560 nivo vs erl: $140,601	NR	The use of a PS or Markov model produced very similar estimates of expected cost, outcomes, and incremental cost-utility from the perspective of Canadian publicly-funded healthcare system.
Matter-Walstra et al., 2016 [21]	Nivo: CHF66,208 ($68,419) Doc: CHF37,618 ($38,874) Nivo with dose reduction: CHF 47,410 ($48,993) Nivo with duration reduction: CHF55,394 ($57,244)	Nivo: 0.69 Doc: 0.53 Nivo with dose reduction: 0.69 Nivo with duration reduction: 0.69	Nivo vs doc: CHF177,478 ($183,406) Nivo (PD-L1≥1%) vs doc: CHF133,267 ($137,718) Nivo (PD-L1≥1%) vs nivo: CHF65,774 ($67,971) Nivo (PD-L1≥10%) vs doc: CHF124,891 ($129,062) Nivo (PD-L1≥10%) vs nivo: CHF37,860 ($39,125) Nivo with dose reduction vs doc: CHF60,787 ($62,817) Nivo with reduced duration vs doc: CHF110,349 ($114,035)	CHF100,000 ($103, 340)	Nivo is not cost-effective compared with docetaxel for non-squamous NSCLC from the perspective of Swiss health care system. However, nivo is cost-effective by dose reduction and increased PD- L1 threshold.
Aguiar et al., 2017 [22]	SCC (PD-L1 unselected): nivo: $104,453 doc: $39,516 non-SCC (PD-L1 unselected): nivo: $100,791 doc: $46,856 All histology (PD-L1≥1%): pembro: $82,201 doc: $48,182 All histology (PD-L1 unselected): atezo: $122,155; doc: $45,864	SCC (PD-L1 unselected): nivo: 0.82 doc: 0.40 non-SCC (PD-L1 unselected): nivo: 0.87 doc: 0.59 All histology (PD-L1≥1%): pembro:0.92; doc: 0.57 All histology (PD-L1 unselected): atezo: 0.90; doc: 0.54	Nivo vs doc (SCC, PD-L1 unselected): $155,605 Nivo vs doc (non-SCC, PD-L1 unselected): $187,685 Pembro vs doc (all histology, PD-L1≥1%): $98,421 Atezo vs doc (all histology, PD-L1≥1%): $215,802	$100,000	Atezo is not cost-effective; pembro is cost-effective; although not at baseline, nivo is cost-effective by increased PD-L1 threshold. The use of PD-L1 expression as a biomarker increases cost-effective of immunotherapy from the perspective of US Medicare system.
Huang et al., 2017 [24]	Pembro: $297,443 Doc: $136,921	Pembro: 1.71 Doc: 0.76	Pembro vs doc: $168,619	$171,660	Pembro is cost-effective compared with docetaxel in pre-treated advanced NSCLC patients with PD-L1 ≥ 50% from the perspective of US third-party payer.
Gao et al., 2019 [31]	PS model: nivo: A$137,935 ($96,763) doc: A$19,257 ($13,509) Markov model: nivo: A$100,236 ($70,316) doc: A$22,534 ($15,808)	PS model: nivo: 1.06 doc: 0.46 Markov model: nivo: 1.03 doc: 0.68	Nivo vs doc PS model: A$198,862 ($139347) Markov model: A$220,029 ($154,179)	A$50,000 ($35036)	Nivo is not cost-effective for patients with previously treated advanced or metastatic squamous NSCLC from the perspective of Australian healthcare system.
Liu et al., 2019 [35]	Nivo: $40,599 Doc: $18,338	Nivo: 0.55 Doc: 0.31	Nivo vs doc: $93,307	$28,899 for general regions; $63,564 for affluent regions	Nivo is not cost-effective compared with doc for patients with previously treated advanced NSCLC from the perspective of Chinese healthcare system.
Ondhia et al., 2019 [37]	Atezo: CAD130,563 ($100,541) Doc: CAD45,490 ($35,030) Nivo: CAD134,839 ($103,834)	Atezo: 1.31 Doc: 0.71 Nivo:1.28	Atezo vs doc: CAD142,074 ($109,406) Nivo vs doc: CAD158,875 ($122,343)	$125,000	Atezo is cost-effective compared with doc, and atezo dominated nivo from the perspective of Canadian publicly-funded healthcare system.
**Locally advanced (n = 1)**					
Criss et al., 2019 [29]	Durvalumab consolidation therapy: $201,563 No consolidation therapy: $185,944	Durvalumab consolidation therapy: 2.34 No consolidation therapy: 2.57	Durvalumab consolidation vs no consolidation: $67,421	$100,000	Durvalumab consolidation therapy can be cost-effective for patients with unresectable stage III NSCLC whose disease has not progressed after chemoradiotherapy from the perspective of US Health Care System.

Abbreviations. QALY, quality adjusted life year; ICER, incremental cost-effectiveness ratio; WTP, willingness-to-pay; PD-L1, programmed cell death ligand-1; pembro, pembrolizumab; PBC, platinum-based chemotherapy; US, United States; NHS, National Health System; UK, United Kingdom; SCC, squamous cell carcinoma; gem, gemcitabine; bev, bevacizumab; ptx, paclitaxel; pem, pemetrexed; HK, Hong Kong; NR, not reported; BTS, test-and-treat strategy; chemo, chemotherapy; NSCLC, non-small cell lung cancer; atezo, atezolizumab; BCP, bevacizumab + carboplatin +paclitaxel; ABCP, atezolizumab + bevacizumab + carboplatin + paclitaxel; CP, carboplatin plus pemetrexed; TC, paclitaxel plus carboplatin; nivo, nivolumab; doc, docetaxel; erl, erlotinib; PS, partitioned survival; CHF, Swiss francs; CAD, Canadian dollars.

In the first-line treatment setting, studies were differentiated according to PD-L1 expression levels (PD-L1 ≥ 50%; PD-L1 ≥ 1%; all PD-L1 expression levels). For patients with PD-L1 ≥ 50%, pembrolizumab could be a cost-effective first-line treatment compared with platinum-based chemotherapy from the perspective of US health care system or third-party payer [23, 25], French healthcare system [28] and Hospital Authority in Hong Kong (China) [36], while not cost-effective in the UK [25, 26], France [28] or China [34]. Four studies evaluated the cost-effectiveness of first-line treatments in PD-L1 ≥ 1% patients [32, 38, 40, 41]. Of these, one study indicated that pembrolizumab was cost-effective compared with platinum-based chemotherapy in patients with all PD-L1 expression level (≥ 50%, ≥ 20%, and ≥ 1%) from the US health care perspective [40]. Nevertheless, another study analyzed from the perspective of US third-party public healthcare payer showed that pembrolizumab was cost-effective in patients with PD-L1 ≥ 1%, but not in the subgroup with PD-L1 1~49% [32]. Similarly, She et al. concluded that pembrolizumab was cost-effective in PD-L1 positive patients (PD-L1 ≥ 50%), but not in the PD-L1 ≥ 20% and ≥ 1% populations in the US [38]. Additionally, Zhou et al. assessed the cost-effectiveness of pembrolizumab from Chinese perspective and found that pembrolizumab is not cost-effective compared with PBC regardless of PD-L1 expression level (≥ 50%, ≥ 20%, and ≥ 1%) [41]. Pembrolizumab and atezolizumab are recommended for use in combination with chemotherapy as first-line treatment regardless of PD-L1 expression status based on KEYNOTE 189, KEYNOTE 407 trial and IMpower150 trial [5, 6, 43]. Insinga et al. [27, 33] evaluated the cost-effectiveness of pembrolizumab plus chemotherapy versus chemotherapy and pembrolizumab monotherapy from the US third-party healthcare payer perspective, and found that this combination might be cost-effective in the first-line treatment of both squamous and non-squamous NSCLC cancer. Two studies investigated the cost-effectiveness of atezolizumab, and results showed that addition of atezolizumab to the combination of bevacizumab, carboplatin, and paclitaxel was not cost-effective for patients with metastatic non-squamous NSCLC from the US payer perspective [30, 39].

Apart from the above-mentioned researches about first-line treatments, six studies assessed the second-line treatments. The earliest study was conducted in 2016 in Canada, from a perspective of publicly-funded healthcare system [20]. This study estimated the cost-effectiveness of nivolumab compared with docetaxel or erlotinib in the second-line treatment of advanced squamous NSCLC, and compared the results using partitioned survival and Markov models, demonstrating these two modelling approaches produced very similar estimates of expected cost, outcomes, and ICER. However, no particular willingness-to-pay (WTP) threshold was mentioned in this study. Subsequently, plenty of economic studies around the world also evaluated the cost-effectiveness of second-line therapies for patients with recurrent NSCLC. Three studies, one each from Switzerland, US and China, showed that nivolumab is not cost-effective compared with docetaxel for patients with previously treated advanced NSCLC [21, 31, 35]. Huang et al. [24] found that pembrolizumab is cost-effective versus docetaxel in pre-treated advanced NSCLC patients with PD-L1 ≥ 50% from the US third-party payer perspective. Additionally, atezolizumab was proved to be a cost-effective therapeutic option in Canada for the treatment of patients with advanced NSCLC who progressed after first-line chemotherapy [37]. Given various options for second-line treatment of advanced NSCLC, Aguiar et al. [22] assessed the cost-effectiveness and economic impact of PD-L1 testing and the above-mentioned three second-line ICIs versus docetaxel. Relative to docetaxel, atezolizumab is not cost-effective; pembrolizumab is cost-effective; though nivolumab is not cost-effective in the base case, it can be cost-effective by increased PD-L1 threshold.

In addition, a study evaluated the cost-effectiveness and potential economic implications of durvalumab, which is the first immunotherapy to be approved for adjuvant treatment of patients with unresectable stage III NSCLC who has not progressed after chemoradiotherapy, in the context of the US health care system [29]. Results demonstrated that durvalumab consolidation therapy can be cost-effective versus no consolidation therapy.

### Quality assessment

**Fig 2:**
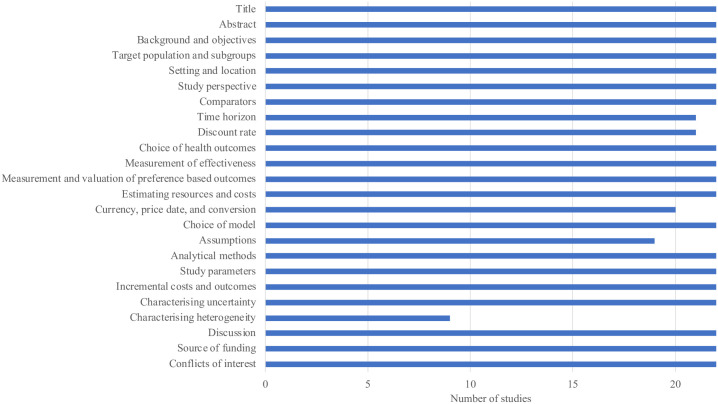
Number of included studies that met each CHEERS criterion.

The included studies had a good reporting quality as per the CHEERS checklist (Fig 2 and S3 File). The least commonly reported item in the included studies was “characterizing heterogeneity,” followed by “assumptions” and “currency, price date, and conversion.” With regard to parameter uncertainty, twenty studies performed one-way sensitivity analysis, twenty studies performed probabilistic sensitivity analysis (PSA), and two studies performed alternative scenario analysis.

## Discussion

In this systematic review, we identified and included 22 studies that evaluated the cost-effectiveness of immune checkpoint inhibitors for treatment of NSCLC. Overall reporting of the identified studies largely met CHEERS recommendations.

In the first-line setting, for patients with PD-L1 ≥ 50%, pembrolizumab appears cost-effective compared with platinum-based chemotherapy in the US and Hong Kong (China), but not in the UK and China. The disparity of the results can be explained by different WTP thresholds across different countries. The conclusions of several studies changed as the WTP threshold increased. Taking various ICER results into account, pembrolizumab could be a cost-effective first-line treatment if WTP threshold set as $100,000/QALY in PD-L1 ≥ 50% patients. The cost-effectiveness of pembrolizumab versus chemotherapy for first-line treatment of NSCLC in PD-L1 ≥1% patients remained obscure. Various factors may explain the difference in the results including variation of drug cost in different countries as well as discrepancy in model parameters. Regardless of PD-L1 expression status, pembrolizumab in combination with chemotherapy can be a cost-effective first-line therapy in the US. On the contrary, addition of atezolizumab to the combination of bevacizumab, carboplatin, and paclitaxel is not cost-effective for patients with metastatic non-squamous NSCLC from the US payer perspective. Even if the threshold is raised to $150,000/QALY, atezolizumab combination cannot be an economic solution in the first-line setting.

In the second-line setting compared with docetaxel, pembrolizumab is cost-effective; though nivolumab is not cost-effective in the base case, it can be cost-effective by increased PD-L1 threshold. It indicates that the use of PD-L1 expression as a biomarker improves the cost-effectiveness of second-line immunotherapy. Results of the cost-effectiveness of atezolizumab second-line treatment remains inconsistent. This is due in part to differences in currencies and healthcare systems between countries and different models adopted for simulation.

In addition, the adoption of durvalumab consolidation therapy after chemoradiotherapy can be cost-effective versus no consolidation therapy for patients with stage III NSCLC, representing an indication that treating with expensive immunotherapy earlier in the course of cancer progression can also provide significant value.

The published systematic reviews of cost-effectiveness studies in immune checkpoint inhibitors are limited. A systematic review by Verma et al. [16] was the first known comprehensive review of cost-effectiveness analysis pertaining to immune checkpoint inhibitors. Since a great many new cost-effectiveness analysis studies were published thereafter, we searched broader databases and included several additional studies for NSCLC treatment. In addition, we summarized and compared more detailed methodological information of the included studies. Subsequently, Addeo et al. [44] evaluated the cost-effectiveness of checkpoint inhibitors plus chemotherapy compared with chemotherapy alone for the first-line treatment of patients with advanced NSCLC. Our results are not directly comparable as this review focused solely on combination of immunotherapy with chemotherapy.

Of note, both abovementioned systematic reviews did not perform quality assessment on the methodological aspects of the included studies. Quality assessment is one of the important steps in developing a good systematic review of cost-effectiveness studies. The most commonly adopted instruments for quality assessment include CHEERS checklist, Quality of Health Economics Studies (QHES) instrument, the Philips and the Drummond checklists, et al. CHEERS checklist was developed by international experts, and jointly endorsed by many journals [19, 45, 46]. A plenty of previous studies have adopted the CHEERS checklist for assessing the quality of published economic studies [47, 48]. Therefore, the quality assessment was conducted using the CHEERS checklists in the present systematic review. Overall, quality of the included studies was high with majority of studies reported strictly according to the CHEERS checklist.

As far as we know, this is the first systematic review to assess the cost-effectiveness of immune checkpoint inhibitors for patients with NSCLC. The majority of published literature reporting cost-effectiveness evaluation pertaining to immunotherapies for NSCLC treatment was included in our analysis, including first-line and second-line treatment for advanced or metastatic disease as well as earlier treatment for stage III carcinoma. Nevertheless, our review has some limitations. First, we restricted our review to studies written in English. In addition, some of the relevant studies may not have been retrieved despite the use of broad search terms in commonly used databases. Finally, the studies included in this review were conducted from different perspectives and countries, and adopting different WTP threshold, leading to difficulties in interpreting the conclusions.

## Conclusion

For patients with NSCLC, immunotherapy can be a cost-effective strategy in several scenarios. A discount of the agents or the use of PD-L1 expression as a biomarker improves the cost-effectiveness of immunotherapy. Future publications of economic evaluation pertaining to immune checkpoint inhibitors for treatment of NSCLC could alter conclusions from this review.

## Supporting information

S1 File PRISMA 2009 checklist. (DOC)

S2 File Search strategy. (DOCX)

S3 File Quality assessment. (XLSX)

S1 Table Overview of the methodology. (DOCX)

## References

[1] Bray F, Ferlay J, Soerjomataram I, Siegel RL, Torre LA, Jemal A. Global cancer statistics 2018: GLOBOCAN estimates of incidence and mortality worldwide for 36 cancers in 185 countries. CA: a cancer journal for clinicians. 2018;68(6):394–424. Epub 2018/09/13. 10.3322/caac.21492 . 30207593

[2] Tun AM, Thein KZ, Thein WL, Guevara E. Checkpoint inhibitors plus chemotherapy for first-line treatment of advanced non-small cell lung cancer: a systematic review and meta-analysis of randomized controlled trials. Future science OA. 2019;5(9):Fso421 Epub 2019/10/15. 10.2144/fsoa-2019-0081 . 31608159 PMC6787520

[3] Planchard D, Popat S, Kerr K, Novello S, Smit EF, Faivre-Finn C, et al Metastatic non-small cell lung cancer: ESMO Clinical Practice Guidelines for diagnosis, treatment and follow-up. Annals of oncology: official journal of the European Society for Medical Oncology. 2019;30(5):863–70. Epub 2019/02/05. 10.1093/annonc/mdy474 . 31987360

[4] Hellmann MD, Paz-Ares L, Bernabe Caro R, Zurawski B, Kim SW, Carcereny Costa E, et al Nivolumab plus Ipilimumab in Advanced Non-Small-Cell Lung Cancer. The New England journal of medicine. 2019;381(21):2020–31. Epub 2019/09/29. 10.1056/NEJMoa1910231 . 31562796

[5] Socinski MA, Jotte RM, Cappuzzo F, Orlandi F, Stroyakovskiy D, Nogami N, et al Atezolizumab for First-Line Treatment of Metastatic Nonsquamous NSCLC. The New England journal of medicine. 2018;378(24):2288–301. Epub 2018/06/05. 10.1056/NEJMoa1716948 . 29863955

[6] Paz-Ares L, Luft A, Vicente D, Tafreshi A, Gumus M, Mazieres J, et al Pembrolizumab plus Chemotherapy for Squamous Non-Small-Cell Lung Cancer. The New England journal of medicine. 2018;379(21):2040–51. Epub 2018/10/04. 10.1056/NEJMoa1810865 . 30280635

[7] Antonia SJ, Villegas A, Daniel D, Vicente D, Murakami S, Hui R, et al Overall Survival with Durvalumab after Chemoradiotherapy in Stage III NSCLC. The New England journal of medicine. 2018;379(24):2342–50. Epub 2018/10/04. 10.1056/NEJMoa1809697 . 30280658

[8] Horn L, Spigel DR, Vokes EE, Holgado E, Ready N, Steins M, et al Nivolumab Versus Docetaxel in Previously Treated Patients With Advanced Non-Small-Cell Lung Cancer: Two-Year Outcomes From Two Randomized, Open-Label, Phase III Trials (CheckMate 017 and CheckMate 057). Journal of clinical oncology: official journal of the American Society of Clinical Oncology. 2017;35(35):3924–33. Epub 2017/10/13. 10.1200/jco.2017.74.3062 . 29023213 PMC6075826

[9] Reck M, Rodriguez-Abreu D, Robinson AG, Hui R, Csoszi T, Fulop A, et al Pembrolizumab versus Chemotherapy for PD-L1-Positive Non-Small-Cell Lung Cancer. The New England journal of medicine. 2016;375(19):1823–33. Epub 2016/10/11. 10.1056/NEJMoa1606774 . 27718847

[10] Fehrenbacher L, Spira A, Ballinger M, Kowanetz M, Vansteenkiste J, Mazieres J, et al Atezolizumab versus docetaxel for patients with previously treated non-small-cell lung cancer (POPLAR): a multicentre, open-label, phase 2 randomised controlled trial. Lancet (London, England). 2016;387(10030):1837–46. Epub 2016/03/14. 10.1016/s0140-6736(16)00587-0 . 26970723

[11] Mariotto AB, Yabroff KR, Shao Y, Feuer EJ, Brown ML. Projections of the cost of cancer care in the United States: 2010–2020. Journal of the National Cancer Institute. 2011;103(2):117–28. Epub 2011/01/14. 10.1093/jnci/djq495 21228314 PMC3107566

[12] Schnipper LE, Davidson NE, Wollins DS, Tyne C, Blayney DW, Blum D, et al American Society of Clinical Oncology Statement: A Conceptual Framework to Assess the Value of Cancer Treatment Options. Journal of clinical oncology: official journal of the American Society of Clinical Oncology. 2015;33(23):2563–77. 10.1200/JCO.2015.61.6706 . 26101248 PMC5015427

[13] Cherny NI, Sullivan R, Dafni U, Kerst JM, Sobrero A, Zielinski C, et al A standardised, generic, validated approach to stratify the magnitude of clinical benefit that can be anticipated from anti-cancer therapies: the European Society for Medical Oncology Magnitude of Clinical Benefit Scale (ESMO-MCBS). Annals of oncology: official journal of the European Society for Medical Oncology. 2015;26(8):1547–73. 10.1093/annonc/mdv249 . 26026162

[14] Romley JA, Delgado A, Shim J, Batt K. The value of novel immuno-oncology treatments. The American journal of managed care. 2018;24(12):e380–e5. Epub 2018/12/27. . 30586486

[15] Yu PP, Eton O, Garrison LP. Challenges in assessing the clinical utility and economic value of immune checkpoint inhibitor therapies of Cancer. Journal for immunotherapy of cancer. 2019;7(1):235 Epub 2019/09/05. 10.1186/s40425-019-0707-9 . 31481112 PMC6724271

[16] Verma V, Sprave T, Haque W, Simone CB 2nd, Chang JY, Welsh JW, et al A systematic review of the cost and cost-effectiveness studies of immune checkpoint inhibitors. J Immunother Cancer. 2018;6(1):128 Epub 2018/11/25. 10.1186/s40425-018-0442-7 . 30470252 PMC6251215

[17] Moher D, Liberati A, Tetzlaff J, Altman DG. Preferred reporting items for systematic reviews and meta-analyses: the PRISMA statement. International journal of surgery (London, England). 2010;8(5):336–41. Epub 2010/02/23. 10.1016/j.ijsu.2010.02.007 . 20171303

[18] Bramer WM, Giustini D, de Jonge GB, Holland L, Bekhuis T. De-duplication of database search results for systematic reviews in EndNote. Journal of the Medical Library Association: JMLA. 2016;104(3):240–3. Epub 2016/07/02. 10.3163/1536-5050.104.3.014 . 27366130 PMC4915647

[19] Husereau D, Drummond M, Petrou S, Carswell C, Moher D, Greenberg D, et al Consolidated Health Economic Evaluation Reporting Standards (CHEERS) statement. Value in health: the journal of the International Society for Pharmacoeconomics and Outcomes Research. 2013;16(2):e1–5. Epub 2013/03/30. 10.1016/j.jval.2013.02.010 . 23538200

[20] Goeree R, Villeneuve J, Goeree J, Penrod JR, Orsini L, Tahami Monfared AA. Economic evaluation of nivolumab for the treatment of second-line advanced squamous NSCLC in Canada: a comparison of modeling approaches to estimate and extrapolate survival outcomes. J Med Econ. 2016;19(6):630–44. Epub 2016/02/07. 10.3111/13696998.2016.1151432 . 26850122

[21] Matter-Walstra K, Schwenkglenks M, Aebi S, Dedes K, Diebold J, Pietrini M, et al A Cost-Effectiveness Analysis of Nivolumab versus Docetaxel for Advanced Nonsquamous NSCLC Including PD-L1 Testing. Journal of thoracic oncology: official publication of the International Association for the Study of Lung Cancer. 2016;11(11):1846–55. Epub 2016/10/25. 10.1016/j.jtho.2016.05.032 . 27311996

[22] Aguiar PN Jr., Perry LA, Penny-Dimri J, Babiker H, Tadokoro H, de Mello RA, et al The effect of PD-L1 testing on the cost-effectiveness and economic impact of immune checkpoint inhibitors for the second-line treatment of NSCLC. Annals of oncology: official journal of the European Society for Medical Oncology. 2017;28(9):2256–63. Epub 2017/06/22. 10.1093/annonc/mdx305 . 28633409

[23] Huang M, Lou Y, Pellissier J, Burke T, Liu FX, Xu R, et al Cost Effectiveness of Pembrolizumab vs. Standard-of-Care Chemotherapy as First-Line Treatment for Metastatic NSCLC that Expresses High Levels of PD-L1 in the United States. Pharmacoeconomics. 2017;35(8):831–44. Epub 2017/06/18. 10.1007/s40273-017-0527-z . 28620848 PMC5548835

[24] Huang M, Lou Y, Pellissier J, Burke T, Liu FX, Xu R, et al Cost-effectiveness of pembrolizumab versus docetaxel for the treatment of previously treated PD-L1 positive advanced NSCLC patients in the United States. J Med Econ. 2017;20(2):140–50. Epub 2016/08/30. 10.1080/13696998.2016.1230123 . 27571538

[25] Georgieva M, da Silveira Nogueira Lima JP, Aguiar P Jr., de Lima Lopes G Jr., Haaland B. Cost-effectiveness of pembrolizumab as first-line therapy for advanced non-small cell lung cancer. Lung Cancer. 2018;124:248–54. Epub 2018/10/01. 10.1016/j.lungcan.2018.08.018 . 30268469

[26] Hu X, Hay JW. First-line pembrolizumab in PD-L1 positive non-small-cell lung cancer: A cost-effectiveness analysis from the UK health care perspective. Lung Cancer. 2018;123:166–71. Epub 2018/08/10. 10.1016/j.lungcan.2018.07.012 . 30089590

[27] Insinga RP, Vanness DJ, Feliciano JL, Vandormael K, Traore S, Burke T. Cost-effectiveness of pembrolizumab in combination with chemotherapy in the 1st line treatment of non-squamous NSCLC in the US. J Med Econ. 2018;21(12):1191–205. Epub 2018/09/07. 10.1080/13696998.2018.1521416 . 30188231

[28] Chouaid C, Bensimon L, Clay E, Millier A, Levy-Bachelot L, Huang M, et al Cost-effectiveness analysis of pembrolizumab versus standard-of-care chemotherapy for first-line treatment of PD-L1 positive (>50%) metastatic squamous and non-squamous non-small cell lung cancer in France. Lung Cancer. 2019;127:44–52. Epub 2019/01/16. 10.1016/j.lungcan.2018.11.008 . 30642550

[29] Criss SD, Mooradian MJ, Sheehan DF, Zubiri L, Lumish MA, Gainor JF, et al Cost-effectiveness and Budgetary Consequence Analysis of Durvalumab Consolidation Therapy vs No Consolidation Therapy After Chemoradiotherapy in Stage III Non-Small Cell Lung Cancer in the Context of the US Health Care System. JAMA Oncol. 2019;5(3):358–65. Epub 2018/12/14. 10.1001/jamaoncol.2018.5449 . 30543349 PMC6439842

[30] Criss SD, Mooradian MJ, Watson TR, Gainor JF, Reynolds KL, Kong CY. Cost-effectiveness of Atezolizumab Combination Therapy for First-Line Treatment of Metastatic Nonsquamous Non-Small Cell Lung Cancer in the United States. JAMA Netw Open. 2019;2(9):e1911952 Epub 2019/09/26. 10.1001/jamanetworkopen.2019.11952 . 31553470 PMC6764123

[31] Gao L, Li SC. Modelled Economic Evaluation of Nivolumab for the Treatment of Second-Line Advanced or Metastatic Squamous Non-Small-Cell Lung Cancer in Australia Using Both Partition Survival and Markov Models. Appl Health Econ Health Policy. 2019;17(3):371–80. Epub 2018/12/12. 10.1007/s40258-018-0452-0 . 30535675

[32] Huang M, Lopes GL, Insinga RP, Burke T, Ejzykowicz F, Zhang Y, et al Cost-effectiveness of pembrolizumab versus chemotherapy as first-line treatment in PD-L1-positive advanced non-small-cell lung cancer in USA. Immunotherapy. 2019;11(17):1463–78. Epub 2019/11/19. 10.2217/imt-2019-0178 31738117

[33] Insinga RP, Vanness DJ, Feliciano JL, Vandormael K, Traore S, Ejzykowicz F, et al Cost-effectiveness of pembrolizumab in combination with chemotherapy versus chemotherapy and pembrolizumab monotherapy in the first-line treatment of squamous non-small-cell lung cancer in the US. Curr Med Res Opin. 2019;35(7):1241–56. Epub 2019/01/17. 10.1080/03007995.2019.1571297 . 30649973

[34] Liao W, Huang J, Hutton D, Li Q. Cost-effectiveness analysis of first-line pembrolizumab treatment for PD-L1 positive, non-small cell lung cancer in China. J Med Econ. 2019;22(4):344–9. Epub 2019/01/17. 10.1080/13696998.2019.1570221 . 30646794

[35] Liu Q, Luo X, Peng L, Yi L, Wan X, Zeng X, et al Nivolumab Versus Docetaxel for Previously Treated Advanced Non-Small Cell Lung Cancer in China: A Cost-Effectiveness Analysis. Clin Drug Investig. 2019 Epub 2019/11/05. 10.1007/s40261-019-00869-3 . 31679121 PMC6989620

[36] Loong HH, Wong CKH, Leung LKS, Dhankhar P, Insinga RP, Chandwani S, et al Cost Effectiveness of PD-L1-Based Test-and-Treat Strategy with Pembrolizumab as the First-Line Treatment for Metastatic NSCLC in Hong Kong. Pharmacoecon Open. 2019 Epub 2019/09/19. 10.1007/s41669-019-00178-7 . 31531842 PMC7248157

[37] Ondhia U, Conter HJ, Owen S, Zhou A, Nam J, Singh S, et al Cost-effectiveness of second-line atezolizumab in Canada for advanced non-small cell lung cancer (NSCLC). J Med Econ. 2019;22(7):625–37. Epub 2019/03/06. 10.1080/13696998.2019.1590842 . 30836031

[38] She L, Hu H, Liao M, Xia X, Shi Y, Yao L, et al Cost-effectiveness analysis of pembrolizumab versus chemotherapy as first-line treatment in locally advanced or metastatic non-small cell lung cancer with PD-L1 tumor proportion score 1% or greater. Lung Cancer. 2019;138:88–94. Epub 2019/10/28. 10.1016/j.lungcan.2019.10.017 . 31655368

[39] Wan X, Luo X, Tan C, Zeng X, Zhang Y, Peng L. First-line atezolizumab in addition to bevacizumab plus chemotherapy for metastatic, nonsquamous non-small cell lung cancer: A United States-based cost-effectiveness analysis. Cancer. 2019;125(20):3526–34. Epub 2019/07/10. 10.1002/cncr.32368 . 31287562

[40] Weng X, Luo S, Lin S, Zhong L, Li M, Xin R, et al Cost-utility analysis of pembrolizumab versus chemotherapy as first-line treatment for metastatic non-small cell lung cancer with different PD-L1 expression levels. Oncol Res. 2019 Epub 2019/10/16. 10.3727/096504019x15707883083132 . 31610828 PMC7851532

[41] Zhou K, Jiang C, Li Q. Cost-effectiveness analysis of pembrolizumab monotherapy and chemotherapy in the non-small-cell lung cancer with different PD-L1 tumor proportion scores. Lung Cancer. 2019;136:98–101. Epub 2019/09/03. 10.1016/j.lungcan.2019.08.028 . 31476529

[42] Mok TSK, Wu YL, Kudaba I, Kowalski DM, Cho BC, Turna HZ, et al Pembrolizumab versus chemotherapy for previously untreated, PD-L1-expressing, locally advanced or metastatic non-small-cell lung cancer (KEYNOTE-042): a randomised, open-label, controlled, phase 3 trial. Lancet (London, England). 2019;393(10183):1819–30. Epub 2019/04/09. 10.1016/s0140-6736(18)32409-7 . 30955977

[43] Gandhi L, Rodríguez-Abreu D, Gadgeel S, Esteban E, Felip E, De Angelis F, et al Pembrolizumab plus Chemotherapy in Metastatic Non-Small-Cell Lung Cancer. The New England journal of medicine. 2018;378(22):2078–92. Epub 2018/04/17. 10.1056/NEJMoa1801005 . 29658856

[44] Addeo A, Banna GL, Metro G, Di Maio M. Chemotherapy in Combination With Immune Checkpoint Inhibitors for the First-Line Treatment of Patients With Advanced Non-small Cell Lung Cancer: A Systematic Review and Literature-Based Meta-Analysis. Front Oncol. 2019;9:264 Epub 2019/05/07. 10.3389/fonc.2019.00264 . 31058078 PMC6478036

[45] Husereau D, Drummond M, Petrou S, Carswell C, Moher D, Greenberg D, et al Consolidated Health Economic Evaluation Reporting Standards (CHEERS) statement. BMJ (Clinical research ed). 2013;346:f1049 Epub 2013/03/27. 10.1136/bmj.f1049 . 23529982

[46] Husereau D, Drummond M, Petrou S, Carswell C, Moher D, Greenberg D, et al Consolidated Health Economic Evaluation Reporting Standards (CHEERS) statement. PharmacoEconomics. 2013;31(5):361–7. Epub 2013/03/27. 10.1007/s40273-013-0032-y . 23529207

[47] Ruiz-Negron N, Menon J, King JB, Ma J, Bellows BK. Cost-Effectiveness of Treatment Options for Neuropathic Pain: a Systematic Review. PharmacoEconomics. 2019;37(5):669–88. Epub 2019/01/15. 10.1007/s40273-018-00761-6 . 30637713

[48] Iannazzo S, Iliza AC, Perrault L. Disease-Modifying Therapies for Multiple Sclerosis: A Systematic Literature Review of Cost-Effectiveness Studies. PharmacoEconomics. 2018;36(2):189–204. Epub 2017/10/17. 10.1007/s40273-017-0577-2 . 29032493

