# Supplementary material for: Cost effectiveness of immune checkpoint inhibitors for treatment of non-small cell lung cancer: A systematic review
Source: PLoS One. 2020 Sep 2;15(9):e0238536. doi: 10.1371/journal.pone.0238536 (PMC7467260; doi:10.1371/journal.pone.0238536)
Supplement: S2 File — (DOCX) [file pone.0238536.s002.docx]

**Appendix 1. Search strategy**

**Pubmed (N = 136)**

1. Carcinoma, Non‐Small‐Cell Lung[Mesh Terms]

2. nsclc[Title/Abstract]

3. lung cancer*[Title/Abstract]

4. lung carcinoma*[Title/Abstract]

5. lung neoplasm*[Title/Abstract]

6. lung tumor*[Title/Abstract]

7. lung tumour*[Title/Abstract]

8. non‐small cell*[Title/Abstract]

9. non small cell*[Title/Abstract]

10. non‐small-cell*[Title/Abstract]

11. #3 OR #4 OR #5 OR #6 OR #7

12. #8 OR #9 OR #10

13. #11 AND #12

14. #1 OR #2 OR #13

15. immunotherapy[MeSH Terms]

16. immunother*[Title/Abstract]

17. immune checkpoint inhibitor[Title/Abstract]

18. programmed cell death-1[Title/Abstract]

19. PD-1[Title/Abstract]

20. programmed cell death ligand-1[Title/Abstract]

21. PD-L1[Title/Abstract]

22. cytotoxic T-lymphocyte associated antigen-4[Title/Abstract]

23. CTLA-4[Title/Abstract]

24. nivolumab[MeSH Terms]

25. nivolumab[Title/Abstract]

26. Opdivo[Title/Abstract]

27. pembrolizumab[Title/Abstract]

28. Keytruda[Title/Abstract]

29. atezolizumab[Title/Abstract]

30. Tecentriq[Title/Abstract]

31. durvalumab[Title/Abstract]

32. Imfinzi[Title/Abstract]

33. ipilimumab[MeSH Terms]

34. ipilimumab[Title/Abstract]

35. yervoy[Title/Abstract]

36. #15 OR #16 OR #17 OR #18 OR #19 OR #20 OR #21 OR #22 OR #23 OR #24 OR #25 OR #26 OR #27 OR #28 OR #29 OR #30 OR #31 OR #32 OR #33 OR #34 OR #35

37. Economics[MeSH Terms]

38. Economic[Title/Abstract]

39. cost[Title/Abstract]

40. cost*[Title/Abstract]

41. #37 OR #38 OR #39 OR #40

42. #14 AND #36 AND #41

**Web of Science (N = 326)**

TS=(non small cell lung cancer OR NSCLC) AND TS=(cost* OR economic*) AND TS=(immunother* OR immune checkpoint inhibitor OR programmed cell death-1 OR PD-1 OR programmed cell death ligand-1 OR PD-L1 OR cytotoxic T-lymphocyte associated antigen-4 OR CTLA-4 OR nivolumab OR pembrolizumab OR atezolizumab OR durvalumab OR ipilimumab)

**Cochrane Library (N = 48)**

#1 MeSH descriptor: [Carcinoma, Non-Small-Cell Lung] explode all trees

#2 (non-small cell lung cancer):ti,ab,kw OR (non-small-cell lung cancer):ti,ab,kw OR (NSCLC):ti,ab,kw OR (non small cell lung cancer):ti,ab,kw

#3 #1 OR #2

#4 MeSH descriptor: [Immunotherapy] explode all trees

#5 (immunotherapy):ti,ab,kw OR (immune checkpoint inhibitor):ti,ab,kw OR (programmed cell death-1):ti,ab,kw OR (PD-1):ti,ab,kw AND (programmed cell death ligand-1):ti,ab,kw OR (cytotoxic T-lymphocyte associated antigen-4):ti,ab,kw OR (CTLA-4):ti,ab,kw OR (nivolumab):ti,ab,kw OR (pembrolizumab):ti,ab,kw OR (atezolizumab):ti,ab,kw OR (durvalumab):ti,ab,kw OR (ipilimumab):ti,ab,kw

#6 #4 OR #5

#7 MeSH descriptor: [Economics] explode all trees

#8 (Economic):ti,ab,kw OR (cost):ti,ab,kw

#9 #7 OR #8

#8 #3 AND #6 AND #9
