# Supplementary material for: Cost effectiveness of immune checkpoint inhibitors for treatment of non-small cell lung cancer: A systematic review
Source: PLoS One. 2020 Sep 2;15(9):e0238536. doi: 10.1371/journal.pone.0238536 (PMC7467260; doi:10.1371/journal.pone.0238536)
Supplement: S1 Table — (DOCX) [file pone.0238536.s004.docx]

**Appendix 2** Overview of the methodology of the included studies

| Study, year | Journal | Country | Perspective | Interventions | Time horizon | Discount rate | Model structure  (cycle length) | Effectiveness source |
| --- | --- | --- | --- | --- | --- | --- | --- | --- |
| **First-line (PD-L1≥50%) (n=6)** | | | | | | | | |
| Huang et al., 2017 | Pharmacoeconomics | USA | US third-party public healthcare payer | (1) Pembrolizumab  (2) PBC | 20 years | 3% | Partitioned survival model  (1 week) | KEYNOTE 024 trial |
| Georgieva et al., 2018 | Lung Cancer | USA | British NHS; US cost perspective | (1) Pembrolizumab  (2) PBC | lifetime | 3% | Markov model  (NR) | KEYNOTE 024 trial |
| Hu et al., 2018 | Lung Cancer | USA | UK healthcare | (1) Pembrolizumab  (2) PBC | lifetime | 3.5% | Markov model  (3 weeks) | KEYNOTE 024 trial |
| Chouaid et al., 2019 | Lung Cancer | France | French healthcare system | (1) Pembrolizumab  (2) PBC | 10 years | 4% | Partitioned survival model  (1 week) | KEYNOTE 024 trial |
| Liao et al., 2019 | J Med Econ | China | Chinese society | (1) Pembrolizumab  (2) PBC | 10 years | 3% | Markov model  (1 month) | KEYNOTE 024 trial |
| Loong et al., 2019 | Pharmacoecon Open | China (Hong Kong) | Hospital Authority in Hong Kong | (1) Pembrolizumab  (2) PBC | 10 years | 3% | Cohort simulation model  (1 week) | KEYNOTE 024 trial |
| **First-line (PD-L1≥1%) (n=4)** | | | | | | | | |
| Huang et al., 2019 | Immunotherapy | USA | US third-party public healthcare payer | (1) Pembrolizumab  (2) PBC | 20 years | 3% | Partitioned survival model  (3 weeks) | KEYNOTE 042 trial |
| She et al., 2019 | Lung Cancer | China | US payer | (1) Pembrolizumab  (2) PBC | 20 years | 3% | Markov mode  (6 weeks) | KEYNOTE 042 trial |
| Weng et al., 2019 | Oncol Res | China | US health care system | (1) Pembrolizumab  (2) PBC | lifetime | 3% | Markov model  (3 weeks) | KEYNOTE 042 trial |
| Zhou et al., 2019 | Lung Cancer | China | Payers in China | (1) Pembrolizumab  (2) PBC | 10 years | 3% | Markov model  (NR) | KEYNOTE 042 trial |
| **First-line (all PD-L1 expression levels) (n=4)** | | | | | | | | |
| Insinga et al., 2018 | J Med Econ | USA | US third-party healthcare payer | (1) Pembrolizumab + CP followed by maintenance pemetrexed  (2) CP followed by maintenance pemetrexed  (3) Pembrolizumab | 20 years | 3% | Cohort simulation model  (1 week) | KEYNOTE 189 trial |
| Criss et al., 2019 | JAMA Netw Open | USA | US healthcare sector | (1) BCP  (2) ABCP  (3) CP  (4) Pembrolizumab + CP  [Base Case 1 (Groups 1 and 2); Base case 2 (Group 1-4)] | NR | 3% | Microsimulation model  (1 month) | IMpower150, KEYNOTE 189 trial |
| Insinga et al., 2019 | Curr Med Res Opin | USA | US third-party healthcare payer | (1) Pembrolizumab+chemotherapy  (2) Chemotherapy | 20 years | 3% | Cohort simulation model  (1 week) | KEYNOTE 407 trial |
| Wan et al., 2019 | Cancer | China | US payer | (1) ABCP  (2) BCP  (3) TC | lifetime | 3% | Markov model  (3 weeks) | IMpower150 trial |
| **Second-line (n=7)** | | | | | | | | |
| Goeree et al., 2016 | J Med Econ | Canada | Canadian publicly-funded healthcare system | (1) Nivolumab  (2) Docetaxel  (3) Erlotinib | 10 years | 5% | Partitioned survival model; Markov model  (4 weeks) | CheckMate 017 trial |
| Matter-Walstra et al., 2016 | J Thorac Oncol | Switzerland | Swiss health care system | (1) Nivolumab  (2) Docetaxel | lifetime | NR | Markov model  (1 month) | CheckMate 057 trial |
| Aguiar et al., 2017 | Ann Oncol | USA | US Medicare system | (1) Docetaxel (not tested for PD-L1 expression)  (2) Several types of immunotherapy (not tested for PD-L1 expression)  (3) Several types of immunotherapy for patients with PD-L1≥1% and docetaxel for patients without PD-L1 expression | lifetime | NR | Decision-analytic model  (NR) | CheckMate 017, CheckMate 057, KeyNote 010, and OAK Study |
| Huang et al., 2017 | J Med Econ | USA | US third-party payer | (1) Pembrolizumab for patients with PD-L1≥50%  (2) Docetaxel | 20 years | 3% | Partitioned survival model  (1 week) | KEYNOTE 010 trial |
| Gao et al., 2019 | Appl Health Econ Health Policy | Australia | Australian healthcare system | (1) Nivolumab  (2) Docetaxel | 6 years | 3% | Partitioned survival model; Markov model  (4 weeks) | CheckMate 017 trial |
| Liu et al., 2019 | Clin Drug Investig | China | Chinese healthcare system | (1) Nivolumab  (2) Docetaxel | lifetime | 3% | Markov mode  (3 weeks) | CheckMate 078 trial |
| Ondhia et al., 2019 | J Med Econ | Canada | Canadian publicly-funded healthcare system | (1) Atezolizumab  (2) Docetaxel  (3) Nivolumab | 10 years | 1.5% | Partitioned survival model  (1 week) | OAK trial |
| **Locally advanced (n=1)** | | | | | | | | |
| Criss et al., 2019 | JAMA Oncol | USA | US healthcare system | (1) Durvalumab consolidation therapy  (2) No consolidation therapy | 5 years | 3% | Markov model  (1 month) | PACIFIC trial |

Abbreviations. PD-L1, programmed cell death ligand-1; USA, the United States of America; SCC, squamous cell carcinoma; PBC, Platinum-based chemotherapy; NHS, National Health System; UK, United Kingdom; NSCLC, non-small cell lung cancer; CP, carboplatin plus pemetrexed; BCP, combination of bevacizumab, carboplatin, and paclitaxel; ABCP, combination of atezolizumab, bevacizumab, carboplatin, and paclitaxel; TC, paclitaxel plus carboplatin; NR, not reported
